# Adventitial inflammation and its interaction with intimal atherosclerotic lesions

**DOI:** 10.3389/fphys.2014.00296

**Published:** 2014-08-08

**Authors:** Mohammadreza Akhavanpoor, Susanne Wangler, Christian A. Gleissner, Grigorios Korosoglou, Hugo A. Katus, Christian Erbel

**Affiliations:** ^1^Department of Cardiology, University of HeidelbergHeidelberg, Germany; ^2^DZHK (German Centre for Cardiovascular Research)Partner Site Heidelberg/Mannheim, Germany

**Keywords:** adventitial inflammation, adventitial tertiary lymphoid organs, atherosclerosis, autoimmunity, vascular smooth muscle cells, macrophages

## Abstract

The presence of adventitial inflammation in correlation with atherosclerotic lesions has been recognized for decades. In the last years, several studies have investigated the relevance and impact of adventitial inflammation on atherogenesis. In the abdominal aorta of elderly *Apoe^−/−^* mice, adventitial inflammatory structures were characterized as organized ectopic lymphoid tissue, and therefore termed adventitial tertiary lymphoid organs (ATLOs). These ATLOs possess similarities in development, structure and function to secondary lymphoid organs. A crosstalk between intimal atherosclerotic lesions and ATLOs has been suggested, and several studies could demonstrate a potential role for medial vascular smooth muscle cells in this process. We here review the development, phenotypic characteristics, and function of ATLOs in atherosclerosis. Furthermore, we discuss the possible role of medial vascular smooth muscle cells and their interaction between plaque and ATLOs.

## Introduction

Atherosclerosis is defined as a chronic inflammatory process of the vascular wall (Nilsson and Hansson, 2008). Coronary artery disease (CAD) and ischemic heart failure as the clinical consequences of atherosclerosis are the major cause of death worldwide, which emphasizes the need for new therapeutic strategies (Go et al., 2013). In addition to affecting the lipometabolism and lowering LDL levels, the manipulation of the chronic immunological process has become of specific interest regarding its therapeutic potential in atherosclerosis. However, despite intensive research investigating the relationship between immunological processes and the development of unstable atherosclerotic lesions, there are still many open questions.

Numerous recent atherosclerosis studies have focused on the intimal atherosclerotic lesion. However, the presence of adventitial inflammation in correlation with atherosclerotic lesions has been recognized for decades (Schwartz and Mitchell, 1962). Just recently, these adventitial inflammatory structures have come into focus, and Gräbner et al. (2009) first characterized some of these cellular compounds as adventitial tertiary lymphoid organs (ATLOs) in old *Apoe^−/−^* mice and established a stage classification (Gräbner et al., 2009).

Tertiary lymphoid organs (TLOs) represent a novel form of ectopic, highly organized immunological structures similar to secondary lymphoid organs like lymph nodes. TLOs have been found to the immunological activation of chronic inflammatory diseases such as chronic infections, transplant rejection, or autoimmune diseases. They develop by lymphoid neogenesis at the site of chronic inflammation with persistent antigen stimulation (Neyt et al., 2012). Although the conditions of TLO induction and their contribution to disease pathology are still not fully understood, they seem to play an important role in local antigen clearance, immune activation and thus affect the course of disease.

When trying to understand the development and function of TLOs during atherogenesis, the special architecture of the vessel wall with three distinct layers has to be noted. Particularly the vascular smooth muscle cells of the lamina media, separating the lamina intima with the atherosclerotic lesion from the lamina adventitia with the associated TLO, are of special interest as they may serve as mediators between the intimal and adventitial processes.

## Tertiary lymphoid organs

Tertiary lymphoid organs develop by lymphoid neogenesis at sites of chronic inflammation with persistent antigen stimulation. This includes chronic infections, transplant rejection or autoimmune diseases (Neyt et al., 2012). This process can take place within or nearby the inflamed tissues (Moyron-Quiroz et al., 2004). In particular, TLO formation could be confirmed in infections like influenza (Moyron-Quiroz et al., 2004; Geurtsvankessel et al., 2009) or Helicobacter pylori (Winter et al., 2010). Furthermore, TLOs are associated with human chronic transplant rejection and could be found during heart, lung, or kidney transplant rejection (Di Carlo et al., 2007; Thaunat et al., 2010; Sato et al., 2011). Similarly, TLO formation could be confirmed in multiple autoimmune diseases: thus, TLOs were found in joints and lung tissue of rheumatoid arthritis patients (Rangel-Moreno et al., 2006), in the thyroid gland of patients with Hashimoto's thyroiditis (Armengol et al., 2001), in the pancreas of patients with diabetes (Kendall et al., 2007) and in the central nervous system of patients with multiple sclerosis (Franciotta et al., 2008). However, it still remains unclear which specific immune conditions lead to TLO induction.

**Figure 1:**
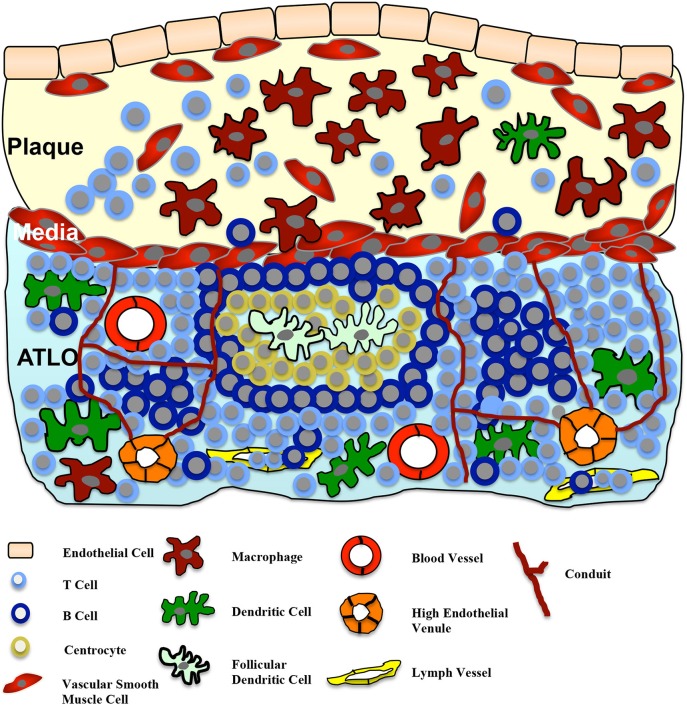
**Adventitial tertiary lymphoid organs (ATLOs) in atherosclerosis**. The cellularity and structure of ATLOs in the diseased vessel wall is presented. ATLOs represent organized accumulation of different lymphoid cells, developed in response to the chronic inflammatory process of atherosclerosis. Stage III ATLOs show T and B cell areas. Germinal centers with follicular dendritic cells surrounded by centrocytes and B cells are present. Lymph vessels and high endothelial venules (HEV) facilitate the recruitment of lymphocytes from the blood into ATLOs. Similar to lymph nodes, ATLOs contain a mesenchymal network of conduits, connecting the lamina media with HEVs in T cell areas. Small molecular weight molecules (such as chemokines and cytokines) can be transported by these conduits. A cross-talk between the plaque and the ATLO via the medial VSMCs is postulated.

TLOs are defined as an accumulation of lymphoid cells and develop in response to chronic inflammation, either due to infection or autoimmunity (Sansonno et al., 2004; Aloisi and Pujol-Borrell, 2006; Kendall et al., 2007; Carragher et al., 2008; Van De Pavert and Mebius, 2010). A more precise definition of TLOs was recently published by Neyt et al. (2012). In contrast to the chronic cellular compound, TLOs can be defined by the following criteria: TLOs (also known as ectopic lymphoid structures) represent highly organized lymphoid cell formations similar to secondary lymphoid organs like lymph nodes or the spleen. The structures of TLOs form a specific, well-organized network including fibroblast reticular cells in the T cell area that warrant optimal interactions of immune cells like antigen presentation, stimulation, and cell differentiation (Fletcher et al., 2010). Similar to lymph nodes, TLOs contain a mesenchymal network promoting lymphocyte homing. Lymph vessels and high endothelial venules (HEV; PNA^+^ or MECA79^+^), specialized for the recruitment of lymphocytes from blood, facilitate lymphocyte migration and are located in the T cell area. Furthermore, structured B cell compartments containing germinal centers and activation-induced cytidinedeaminase (Geurtsvankessel et al., 2009; Perros et al., 2012) as well as T cell sections co-localized with antigen-presenting cells, including follicular dendritic cells, can be found (Fletcher et al., 2010). Figure 1 demonstrates a simplified illustration of the structure of TLOs in the adventitia in correlation to atherosclerotic lesions.

TLOs develop in a similar fashion as secondary lymphoid organs do. The development is orchestrated by various chemokines. Accordingly, elevated levels of homeostatic cytokines and chemokines like CXCL12, CXCL13, CCL20, CCL21, LTα and LTβ were found at the site of chronic or autoimmune inflammation (Carragher et al., 2008). In the early phase, lymphoid tissue inducer cells (LTi cells) expressing lymphotoxin α1β2 (LTα1β2) interact with stromal lymphoid tissue organizer cells (LTo) carrying the lymphotoxin β receptor (LTβR) on their surface thereby inducing lymphoid organogenesis (Mebius, 2003; Muller and Lipp, 2003). Investigation of TLO formation during chronic inflammation or autoimmune processes revealed that several immune cells can also function as LTi cells. McDonald et al. showed that activated B cells expressing LTβ can induce TLO formation in the murine mucosal immune system (McDonald et al., 2005), while Marinkovic et al. identified mature CD3^+^CD4^+^ T cells as potential triggers of TLO development (Marinkovic et al., 2006). In experimental autoimmune encephalomyelitis – a mouse model of multiple sclerosis – Peters et al. demonstrated that Th17 cells also contribute to the formation of TLOs (Peters et al., 2011). Interestingly, IL-17—the distinctive cytokine of Th17 cells—also seems to be important for the formation of inducible bronchus-associated lymphoid tissue (iBALT) by inducing lymphotoxin α-independent expression of CXCL13 in murine lungs (Rangel-Moreno et al., 2011). Besides B and T cells, dendritic cells as the most potent antigen presenting cell type are able to induce TLOs, while the depletion of dendritic cells leads to their disappearance (Geurtsvankessel et al., 2009; Halle et al., 2009; Muniz et al., 2011). After activation, LTo cells express various chemokines and cytokines leading to further recruitment of different lymphocytes. T cells are attracted by CCL19 and CCL21, whereas CXCL13 triggers the recruitment of B cells. The development of LYVE-1^+^ lymph vessels is promoted by the expression of lymphangiogenic growth factors like fibroblast growth factor (FGF), vascular endothelial growth factor (VEGF), or hepatocyte growth factor (HGF). This results in formation of a mesenchymal scaffold for the recruitment of T and B cells, follicular dendritic cells, and fibroblasts, leading to the development and progression of lymphatic organogenesis.

The role of TLOs within the immune system is double-edged: on the one hand, TLOs may help to eliminate pathogens during infections like influenza (Moyron-Quiroz et al., 2004) and thereby protect the organism against foreign antigens. On the other hand, their development especially during autoimmunity may lead to ongoing activation of predominantly autoimmune T cells and subsequently disturb the balance between pro- and anti-inflammatory factors leading to uncontrolled inflammation. This disequilibrium may in turn lead to tissue destruction and organ damage. The development and function of TLOs in general has been excellently reviewed by Weih et al. (2012).

## History of adventitial inflammation and atherosclerosis

In 1962, Schwartz et al. described for the first time a cellular infiltration of the adventitia of human arteries associated with atherosclerotic plaques (Schwartz and Mitchell, 1962). Although this adventitial accumulation of cells has been recognized decades ago, the main focus of atherosclerosis research has been on plaque development within the intimal layer. Nonetheless, some studies also addressed the role of the adventitial inflammatory process in atherosclerosis. Thus, in 1981 Parums et al. suggested a correlation between the degree of adventitial inflammation and the severity of the atherosclerotic lesion (Parums and Mitchinson, 1981). In 1985, Kohchi et al. quantitatively analyzed adventitial inflammation in coronary arteries of patients after fatal myocardial infarction compared to patients who died due to non-cardiac causes. This study pointed toward a possible relevance of adventitial infiltration of immune cells with unstable CAD (Kohchi et al., 1985). In the following years, several studies further characterized the cellular composition of these adventitial cell aggregations (Wick et al., 1997; Houtkamp et al., 2001; Zhao et al., 2004; Moos et al., 2005; Galkina et al., 2006; Watanabe et al., 2007). T and B cells were shown to represent the main cell type within these structures. In 2005, Moos et al. found an age-dependent increase of adventitial T cell inflammation in *Apoe^−/−^* mice fed a normal chow diet (Moos et al., 2005). T and B cell clusters as well as follicle-like structures were identified in abdominal aortas of older *Apoe^−/−^* mice (Moos et al., 2005).

In 2009, Gräbner et al. were the first to show that these adventitial cellular compounds can advance into tertiary lymphoid organs in the adventitia of abdominal aortas of *Apoe^−/−^* mice (Gräbner et al., 2009). In this study, the authors precisely analyzed the cellular composition and structure of ATLOs in the adventitia of abdominal aortas of *Apoe^−/−^* mice and established an ATLO stage classification. ATLO neogenesis was found in the adventitia of abdominal aortas of some 32 weeks old *Apoe^−/−^* mice on chow diet and occurred in >70% of mice afflicted with atherosclerosis at 78 weeks (Gräbner et al., 2009). They postulated that ATLOs appear in advanced late-stage atherosclerosis. Interestingly, ATLOs always appeared in adventitial segments of the abdominal aorta affected by atherosclerotic lesions and the lesion size correlated with the ATLO size (Gräbner et al., 2009). Because of the co-localization of adventitial inflammatory structures and ATLOs with atherosclerotic lesions, it was tempting to suggest an interaction between both compartments in the vascular wall. The vascular smooth muscle cells in the lamina media separating both compartments may coordinate this crosstalk as we will discuss later in this review. Table 1 gives an overview over the study's investigating adventitial inflammation in atherosclerosis.

**Table 1:** **Adventitial inflammation in relation to atherosclerosis**.

**References**	**Organism**	**Investigated vessel**	**Main result**
Schwartz and Mitchell, 1962	Human	Aorta, coronary artery, cervical and iliac artery	Adventitial cellular infiltration associated to atherosclerotic plaques
Parums and Mitchinson, 1981	Human	Coronary artery	Correlation between adventitial inflammation and atherosclerotic plaque
Kohchi et al., 1985	Human	Coronary artery	Relevance of adventitial inflammation to unstable CAD
Houtkamp et al., 2001	human	Aorta	Similarity of adventitial lymphoid infiltrates and mucosa associated lymphoid tissue MALT in advanced atherosclerosis
Moos et al., 2005	*Apoe^−/−^* mice	Abdominal aorta	Formation of inflammatory follicle-like structures in the abdominal aorta of old *Apoe^−/−^* mice
Watanabe et al., 2007	Human	Coronary artery	Formation of small lymph follicle-like structures in the adventitia of coronary arteries
Gräbner et al., 2009	*Apoe^−/−^* mice	Abdominal aorta	Identification of ATLOs in the abdominal aorta of old *Apoe^−/−^* mice

## Do adventitial inflammatory processes represent TLOs?

**Figure 2:**
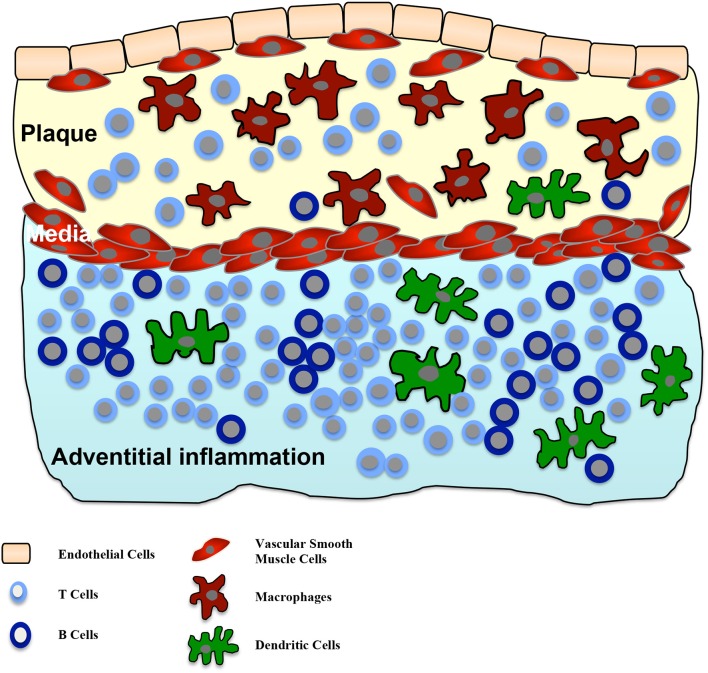
**Adventitial inflammation in atherosclerosis**. Intimal atherosclerotic lesions are frequently accompanied by an inflammatory process in the adjacent adventitia. This inflammatory process is characterized by infiltration of T cells, B cells and dendritic cells in the adventitia. In some cases T and B cell aggregations can be seen. There is a correlation between the size of the intimal lesion and the adventitial structures and a crosstalk between both compartments is suggested.

Human adventitial inflammation was firstly described in 1962 (Schwartz and Mitchell, 1962). The frequency of the inflammatory compound next to atherosclerotic lesions varies between publications, several studies found percentages between 70 and 100% (Houtkamp et al., 2001; Watanabe et al., 2007). Within the adventitial inflammatory compounds, primarily T cells, but also some B cells and dendritic cells are present. Later-stage organized inflammatory compartments further contain lymph follicles (Watanabe et al., 2007). But whether adventitial inflammatory compounds represent ATLOs remains unknown. In addition, it can be speculated whether these adventitial compounds may transform to ATLOs, but in humans studies demonstrating ATLOs are still lacking. Figure 2 illustrates the composition of adventitial inflammation in atherosclerosis.

However, in mice Gräbner et al. convincingly showed that adventitial inflammation at least partly represent ATLOs. ATLO neogenesis occurred in mice older than 32 weeks on chow diet in the abdominal aorta (Gräbner et al., 2009). But the detailed definition of TLOs listed above is limited by the fact that cellular inflammatory compartments may not fulfill all criteria, but still function as TLO (Neyt et al., 2012). In addition, it is not known whether all adventitial cellular compartments can be defined as ATLOs. Thus, further studies are needed to investigate the possible role of adventitial inflammatory compounds in atherosclerosis and the potential steps of the differentiation of adventitial inflammatory compounds toward ATLOs. In addition, it needs to be elucidated whether the facts of TLO in the abdominal aorta are transferable to other parts of the vascular tree.

## Medial vascular smooth muscle cells: the link between intimal atherosclerotic lesions and adventitial inflammation?

**Figure 3:**
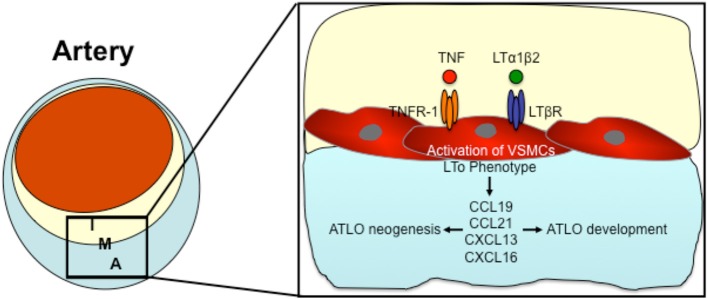
**The possible role of VSMCs in the formation of Adventitial tertiary lymphoid organs (ATLOs)**. Pro-inflammatory cytokines (such as TNF and LTα1β2) activate VSMCs by TNFR-1 or LTßR signaling thereby inducing an LTo phenotype in VSMCs. Activated VSMCs express lymphorganogenic chemokines such as CCL19, CCL21, CXCL13, and CXCL16, thereby orchestrating ATLO neogenesis and developement. I, Intima; M, Media; A, Adventitia.

Based on the above mentioned data, there seems to be a communication between the atherosclerotic lesion in the intima and TLOs in the adventitia. Since both compartments of the arterial wall are separated by vascular smooth muscle cells (VSCMs) in the lamina media, it is likely that these VSMCs coordinate the communication between both compartments. The direct traffic of inflammatory cells through the smooth muscle layer of the lamina media is limited (Dal Canto et al., 2001). As (Gräbner et al., 2009) demonstrated, medial VSMCs surrounded by intimal plaque and ATLOs were able to express the chemokines CXCL13 and CCL21 through the LTßR-dependent signaling pathway (Gräbner et al., 2009). CXCL13 and CCL21 are both lymphorganogenic chemokines that are involved in the formation of secondary and tertiary lymphoid organs (Luster, 1998; Luther et al., 2000; Chen et al., 2002; Cupedo and Mebius, 2005; Charo and Ransohoff, 2006). Gräbner et al. suggested that activated VSCMs underlying atherosclerotic lesion develop properties of LTo cells and thereby induce ATLO neogenesis (Gräbner et al., 2009). LTos are described as mesenchymal cells that control embryonic lymph node development (Luther et al., 2002; Mebius, 2003; Carragher et al., 2008). In 2010, Lotzer et al. supported the hypothesis that VSMCs may differentiate into LTo cells (Lotzer et al., 2010). Simultaneous stimulation of mouse aortic smooth muscle cells by TNF-α and LTßR signaling induced expression of several homeostatic and lymphorganogenic chemokines, which are thought to promote the recruitment of leukocytes to the adventitia and thereby enable the formation of ATLOs (Lotzer et al., 2010). Furthermore, Gräbner et al. could recognize lymph node like conduits connecting medial VSMCs to ATLOs (Gräbner et al., 2009); low molecular weight molecules can be transported into ATLOs via these conduits (Gräbner et al., 2009). In lymph nodes, similar conduits connect afferent lymph vessels and high endothelial venules, coordinating the transport of antigens into lymph nodes (Itano and Jenkins, 2003; Sixt et al., 2005). During the development of secondary lymphoid organs, LTo cells are activated by lymphoid tissue inducer cells (Mebius, 2003). In 2014, Guedj et al. demonstrated the capability of M1 macrophages to act as LTi cells triggering the differentiation of VSMCs into LTo cells (Guedj et al., 2014). They suggest that the interaction between M1 macrophages and VSMCs may induce ATLO formation (Guedj et al., 2014). Taken together, all data published up to date suggest that VSMCs may be involved in ATLO development and interaction of atherosclerotic lesions with ATLOs. Figure 3 demonstrates the possible role of VSMCs in the lamina media between ATLOs and atherosclerotic lesions in the development of ATLOs.

## Conclusions and future directions

Atherosclerosis is a chronic (auto)immune process. To better understand the underlying mechanisms, one should study the entire vessel wall, including inflammatory compounds that develop in the adventitia. Over the past few years, we have gained insight into the structure of these adventitial cellular compartments. Recent studies convincingly show that murine adventitial inflammation at least in part represents different stages of ATLOs. In addition, there seems to be a crosstalk between the intimal lesion and the adventitial cellular compartment through VSMCs.

On the basis of recent studies, it is tempting to postulate a role for adventitial inflammation/ATLOs in the atherogenesis, however, the exact significance of these structures remains enigmatic. A number of questions will have to be addressed by future research: do the adventitial cellular compounds next to atherosclerotic lesions in humans represent ATLOs? What are the exact conditions under which adventitial inflammation advances to ATLOs? Finally, are adventitial inflammatory compartments/ATLOs relevant for human atherogenesis and is their presence associated with the development of unstable lesions? Thus, further studies are needed to clarify the significance of adventitial inflammation. This knowledge significantly advance our understanding of chronic (auto)immune process of the entire vessel wall.

One way, by which the role of ATLOs in human atherosclerosis may be elucidated, may be the application of novel diagnostic imaging tools. For example, specific contrast agents in combination with dedicated magnetic resonance imaging (MRI) pulse sequences nowadays enable the visualization of vessel wall thickness and macrophage-rich inflammation within atherosclerotic plaques (Kim et al., 2007; Korosoglou et al., 2008). These imaging approaches may help to understand the underlying pathophysiologic mechanisms and associations between intimal lesions and adventitial inflammation in future studies.

### Conflict of interest statement

The authors declare that the research was conducted in the absence of any commercial or financial relationships that could be construed as a potential conflict of interest.
